# Rare Type Cranial Postauricular Pilonidal Sinus: A Case Report and Brief Review of Literature

**DOI:** 10.1155/2017/5791972

**Published:** 2017-01-03

**Authors:** Sebahattin Destek, Vahit Onur Gul, Serkan Ahioglu

**Affiliations:** ^1^Department of General Surgery, Bezmialem Vakif University School of Medicine, Istanbul, Turkey; ^2^Edremit State Hospital General Surgery Department, Edremit, 10300 Balikesir, Turkey; ^3^Edremit State Hospital Biochemistry Department, Edremit, 10300 Balikesir, Turkey

## Abstract

A pilonidal sinus is a chronic inflammatory process, the etiology of which is still not fully elucidated. It frequently presents at the sacrococcygeal region (typical) and rarely at extrasacrococcygeal (atypical) locations, including the interdigital, axillary, inguinal, and umbilical regions, as well as the neck, face, and scalp. A 46-year-old man presented with a thirty-year history of a slowly growing nodule on the postauricular area of the scalp. The lesion was excised and diagnosed as a pilonidal sinus based on the clinical and histological findings. The purpose of this review is to report the unusual occurrence of a pilonidal sinus on the scalp and to review the literature regarding this particular location of occurrence.

## 1. Introduction

Pilonidal sinuses (PS) were first described as cysts containing hair by Herbert Mayo in 1833. After that, in 1847, AW Anderson described the PS as a hair-containing ulcer. R. M. Hodges firstly used the phrase pilonidal cyst to describe the condition in 1880. Pilonidal means “nest of hair” and is derived from the Latin words for hair (pilus) and nest (nidus) [1]. PS are a chronic inflammatory condition caused by penetration of hair pieces into the skin. PS is most seen in the sacrococcygeal region, but it may also be seen in atypical areas where hair is able to penetrate the skin, such as the interdigital, umbilical, and axillary regions, as well as the breasts, neck, scrotum, vulva, jaw, face, nose, ears, and scalp [2]. In this article, we present a rare case of PS located at the left postauricular area of the scalp. We hereby reviewed the current literature with emphasis on the pathogenesis and developing aspects of treatment.

## 2. Case Report

A 46-year-old man was admitted to our clinic with a history of a slow-growing nodule lasting thirty years and located behind the ear. He had no complaints of pain or discharge and had no chronic disease history. However, there was a history of trauma such as bumping with stones during his childhood. Examination revealed a painless, soft, slightly tender subcutaneous nodule with normal skin appearance and limited by about 3 cm in diameter, located on the left temporal bone.

The patient's blood counts, C-Reactive Protein (CRP), and erythrocyte sedimentation rates were normal. Two-way direct radiography of the cranium showed no pathology.

**Figure 1:**
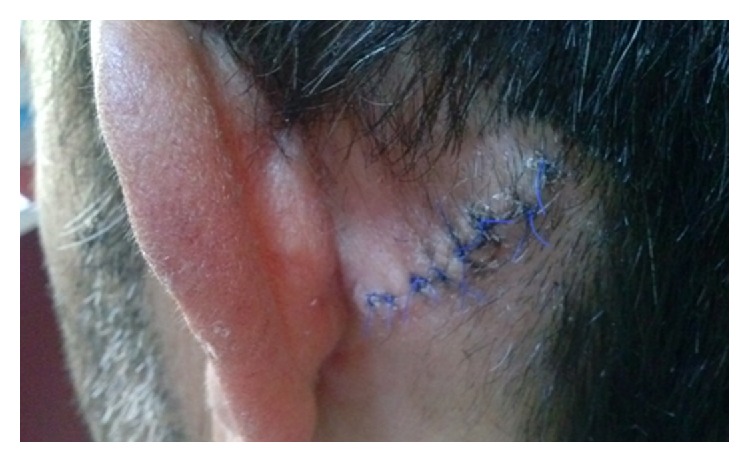
Postauricular sinus excision location.

**Figure 2:**
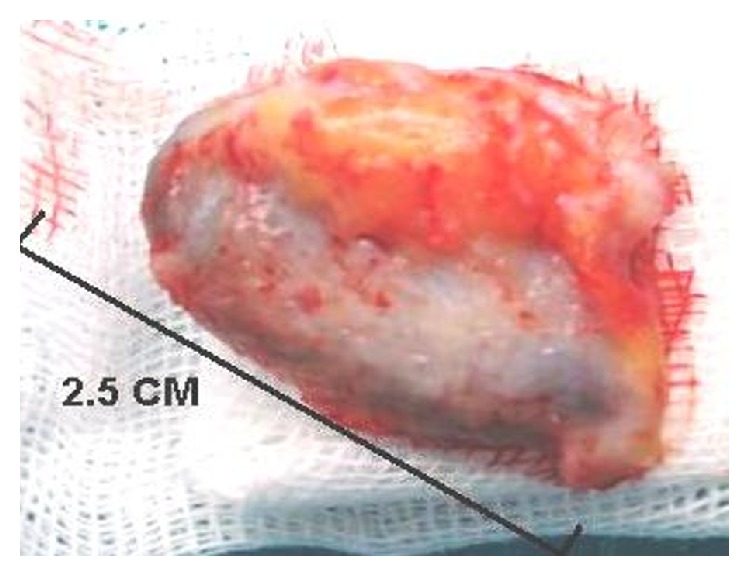
Postauricular pilonidal sinus specimen.

The mass was totally excised under local anesthesia, with its elliptical capsule located over periosteum of the temporal bone (Figures 1-2).

**Figure 3:**
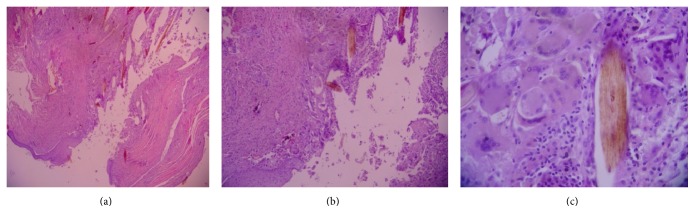
Hematoxylin-eosin stained slides of a specimen of pilonidal sinus obtained from the scalp of a 46-year-old patient. (a) 40, (b) 100, and (c) 400 times magnification images.

It was identified as a pilonidal sinus according to the pathology report findings of a free hair shaft into the dermis and foreign body type of intense inflammatory reaction (Figure 3). There was no recurrence for 2 years of follow-up after treatment.

## 3. Discussion

The first reported case of extrasacrococcygeal pilonidal sinus (ESPS) was located in the interdigital region and reported in 1942 [2]. Similarly, the first ESPS located at the scalp was reported in 1972 by Moyer [3]. PS incidence is 26/100,000 and 97.8% percent of PS are observed in the sacrococcygeal region, with 2.2% observed at the extrasacrococcygeal locations. Pilonidal sinuses located on the scalp are very rare, with only about 0.2% occurrence [2].

PS typically occur in the second or third decades of life and attain peak incidence between ages 15 and 25 years; as expected, 80% of patients are male [1, 3]. It is rarely seen in patients over 40 years, and it occurs 3-4 times more often in men when compared with women [4]. The disease is more often seen in the Mediterranean region; it is rarely encountered in Africans or Asians. It is seen much more in Caucasians when compared to blacks [1, 2]. The average age reported for PS disease in the literature is 25.8 years. Our patient was 46 years old.

Although the aetiology of PS remains unclear and the cause is still not completely understood, in the past, it was focused on embryonic malformations and congenital etiology consisting of teratogenic factors [1].

PS has been observed in 12% of relatives of patients [1]. Nowadays, it is mostly accepted as an acquired skin disease [4]. Total testosterone and prolactin hormone elevations, mechanical trauma from bumps, frictions and shaving, and infections such as folliculitis are among the associated causes [4, 5].

It is considered that local and repeated minor trauma of hairy areas is the major pathogenic cause of PS [5]. When there is no reasonable explanation and the lesion exists from birth, it should be considered as congenital etiology [4].

PS occur with a combination of hair, congenital cleft lesions, skin scars, and hormonal and hygiene problems [5]. Wide intergluteal depth, personal hygiene habits, long periods of sitting, increased body mass index, and genetics have been reported as predisposing factors for PS [1, 4].

*Scalp PS.* Despite these basic etiological causes, 70% of cases of scalp PS are of unknown etiology [5, 6]. Trauma history is reported in about 30% of cases reported in the literature, and, in some cases, this trauma history may date back longer than 20 years [4], such as in the present case report.

Disease sometimes may be latent for years without any signs, while sometimes it may be manifested by acute abscess [1]. In the presence of acute abscess, there is pain, redness, and swelling. Most abscesses spontaneously recover, while others may require medical treatment.

Some PS manifestations bypass the acute abscess stage and instead move directly from the lighter form to the chronic phase of the disease. Chronic cases are manifested by discharge with occurrence of complex sinus and fistula tracts.

Scalp PS usually occurs in the back of the scalp [5, 6]. Scalp PS frequently demonstrates itself as a quiet and painless growing mass [7]. However, sometimes it can be infected and present with pain, abscesses, and fistulas on the scalp [6, 7]. In our case, there was a silent grown mass complaint in the back of left ear on the scalp, but no pain.

Sebaceous cysts, lipoma, fibroma, fibrous dysplasia, epidermoid cysts, dermoid cysts, lymphadenopathy, hidradenitis suppurativa, eosinophilic granuloma, Ewing's sarcoma, osteoma, and calvaria should be considered at differential diagnosis. Ultrasound, cranial tomography (CT) or magnetic resonance imaging can be used for diagnosis in conjunction with physical examination [1, 3]. In order to detect the presence of infection, complete blood count tests, abscess culture, and antibiogram should be performed [4]. If it is necessary for clinical diagnosis, a fine needle or core biopsy can be performed. Sedimentation, CRP, blood count tests, and direct radiography were analysed in our case, and a biopsy was not needed for diagnosis.

PS complications are mostly abscess and fistula formation; sometimes osteomyelitis and very rarely malignant transformation may be seen in long-term treatment patients [1, 4]. Development of squamous cell carcinoma and verrucous carcinoma has been reported in some cases in the literature [1, 4]. Therefore, it is necessary to proceed with caution in the diagnosis and choice of treatment. In our case, there was no complication related to PS after two years of follow-up.

For surgical treatment of scalp PS, lesions are often totally excised together with their capsule and closed primarily on the scalp [5, 8]. In large-scale and noninfected PS cases, according to the case condition, convenient flap surgery can be performed in the same session to close the excision area. The form of sinus flap to apply should be determined according to shape and complexity of the defect and considering how the space left empty after excision will be filled [8, 9].

Early surgical complications of PS include infection, hematoma, seroma, and decomposition. Late complications are numbness, pain, itching, recurrence, and cosmetic problems [1, 4].

After treatment, all patients should be followed up for recurrence [8, 10]. For the purpose of avoiding recurrence, there should not be any PS tissue left after the surgery, and there should also be no dead space remaining or wound infection which affects the recovery process [8, 9]. However, no method of treatment can entirely prevent recurrence and relapse; rates or relapse vary between 2 and 40% [8, 10].

Scalp PS with infection is rare, and the recurrence risk is very low after excision. Recurrence was observed in one case in the literature [5]. In cases of a developing acute pilonidal abscess or fistula formation, drainage with debridement and abscess cultures should be performed [10].

Aerobic and/or anaerobic bacteria can reproduce in abscess cultures. According to culture and antibiogram results, patients should be given an appropriate antibiotic agent to prevent dissemination to surrounding tissue. Antibiotic treatment selection can be changed according to the results of culture [8, 10]. Daily dressings should be performed. When malignancy is determined, extensive resection with flap reconstruction and regional lymph node dissection are performed in accordance with the principles of oncologic surgery. After surgery, radiotherapy and chemotherapy should be applied. However, in these cases, the relapse rate is high and the prognosis is poor [11].

In our case, the totally excised mass was examined by pathology and precisely diagnosed as a pilonidal sinus. Follow-up examinations performed 2 years later showed no complications and no recurrence.

As a result, during the process of diagnosing scalp masses, scalp PS should be considered. Although scalp PS are rare, differential diagnosis is crucial. For treatment, proper surgical techniques should be performed and monitoring should be focused on postsurgical complications and recurrence.
